# Transcriptome Analysis and Metabolic Profiling of Green and Red Mizuna (*Brassica rapa* L. var. *japonica*)

**DOI:** 10.3390/foods9081079

**Published:** 2020-08-08

**Authors:** Chang Ha Park, Sun Ju Bong, Chan Ju Lim, Jae Kwang Kim, Sang Un Park

**Affiliations:** 1 Department of Crop Science, Chungnam National University, 99 Daehak-ro, Yuseong-gu, Daejeon 34134, Korea; parkch804@gmail.com (C.H.P.); asop_258@naver.com (S.J.B.); 2 Asia Seed Co., Ltd., 109-35, 518 Beon-gil, Gyeongchungdae-ro, Janghowon-eup, Icheon-si, Gyeonggi-do 17414, Korea; cabbage@asiaseed.co.kr; 3 Division of Life Sciences and Bio-Resource and Environmental Center, Incheon National University, Incheon 22012, Korea

**Keywords:** mizuna, transcriptome, metabolome, glucosinolates, anthocyanins, phenolics

## Abstract

Mizuna (*Brassica rapa* L. var. *japonica*), a member of the family Brassicaceae, is rich in various health-beneficial phytochemicals, such as glucosinolates, phenolics, and anthocyanins. However, few studies have been conducted on genes associated with metabolic traits in mizuna. Thus, this study provides a better insight into the metabolic differences between green and red mizuna via the integration of transcriptome and metabolome analyses. A mizuna RNAseq analysis dataset showed 257 differentially expressed unigenes (DEGs) with a false discovery rate (FDR) of <0.05. These DEGs included the biosynthesis genes of secondary metabolites, such as anthocyanins, glucosinolates, and phenolics. Particularly, the expression of aliphatic glucosinolate biosynthetic genes was higher in the green cultivar. In contrast, the expression of most genes related to indolic glucosinolates, phenylpropanoids, and flavonoids was higher in the red cultivar. Furthermore, the metabolic analysis showed that 14 glucosinolates, 12 anthocyanins, five phenolics, and two organic acids were detected in both cultivars. The anthocyanin levels were higher in red than in green mizuna, while the glucosinolate levels were higher in green than in red mizuna. Consistent with the results of phytochemical analyses, the transcriptome data revealed that the expression levels of the phenylpropanoid and flavonoid biosynthesis genes were significantly higher in red mizuna, while those of the glucosinolate biosynthetic genes were significantly upregulated in green mizuna. A total of 43 metabolites, such as amino acids, carbohydrates, tricarboxylic acid (TCA) cycle intermediates, organic acids, and amines, was identified and quantified in both cultivars using gas chromatography coupled with time-of-flight mass spectrometry (GC-TOFMS). Among the identified metabolites, sucrose was positively correlated with anthocyanins, as previously reported.

## 1. Introduction

Plant species of the family Brassicaceae, previously called Cruciferae, have long been considered health-boosting vegetables and are commonly cultivated worldwide because they provide high amounts of dietary minerals, amino acids, carbohydrates, fatty acids, dietary fibers, vitamins (tocopherol, ascorbate, and folate), and bioactive molecules, including glucosinolates, phenylpropanoids, anthocyanins, and carotenoids. Furthermore, they have been commercially important as a main element of the daily human diet as well as a source of vegetable oil for the food and biodiesel industry [1,2,3,4,5].

Glucosinolates, found mainly in Brassicaceae plants, are a variable and diverse group of natural products, representing a wide range of more than 200 naturally occurring secondary metabolites [6] and share a general structure composed of a D-thioglucose moiety, a sulfonated oxime group, and a flexible side-chain derived from phenylalanine (Phe), methionine (Met), tryptophan (Trp), and branched-chain amino acids [5]. Subsequently, an endogenous enzyme, namely myrosinase, hydrolyzes glucosinolates to a variety of biologically active compounds, including vinyl oxazolidinethiones, nitriles, epithionitriles, isothiocyanates, thiocyanates, and indoles [5]. These compounds are involved in plant defense [5] and exhibit a wide range of pharmaceutical and biological activities associated with the reduced risk of breast, liver, lung, stomach, pancreas, colon, rectum, prostate, and colorectal cancers [1,4,5,7,8].

Phenylpropanoids, including lignin, flavonoids, coumarins, and a variety of small phenolic molecules, are natural products with biological properties that contribute to plant protection against environmental, physical, and biological stresses, such as a microbial, insect, and herbivore attacks, plant-plant interactions, excessive exposure to visible (light) and ultraviolet radiation (UV), drought, and low and high temperatures [9,10]. Moreover, consuming Brassicaceae family plants is potentially beneficial to human health because they contain a variety of phenylpropanoids, which have anti-allergic, anticancer, anti-estrogenic, antioxidant, vascular and cytotoxic antitumor, and antimicrobial properties [1,4,11,12].

Anthocyanins, belonging to the flavonoid group, are plant pigments mainly responsible for color development in different plant organs, and their basic structures consist of an anthocyanidin (also called aglycon) attached to sugar moieties. Additionally, when linked to organic acids, they are known as acylated anthocyanins [13]. Among the six major anthocyanidins (cyanidin, delphinidin, malvidin, pelargonidin, peonidin, and petunidin) present in Brassicaceae vegetables, cyanidin is the most ubiquitous [11]. Anthocyanins are involved in many plant physiological processes such as photo-protection [13] and defense against environmental and biological stresses [14]. In addition, they play a role in color development in flower petals, fruit peels, and seed coats, thereby facilitating pollination and seed dispersal [15,16].

Mizuna (*B. rapa* L. var. *japonica*), commonly cultivated in Japan, is a leafy vegetable characterized by dense leaf rosettes, pinnatisect-type leaves, and long and thin petioles [17]. This plant has been used as a salad vegetable and has been shown to have human health-beneficial properties, such as antioxidant [18,19] and anti-inflammatory effects [19]. Though mizuna is an important crop, there has been no research on a comprehensive description of the metabolic difference between the green and red mizuna through transcriptome analysis based on next-generation sequencing (NGS) and mass spectrometry-based metabolic profiling. Great progress in NGS technology enables an approach to whole-transcriptome sequencing rather than whole-genome sequencing, such that an RNA-seq technique provides functional information from the transcripts of the genome and is regarded as a cost-effective approach [20]. Among the well-known NGS technologies, Illumina Solexa GA, one of the “short-read” (36–72 bp) sequencing technologies, has been successfully used to perform the whole-transcriptome analysis of the Brassicaceae species, such as *Brassica. rapa* [21], *B. oleracea* L. *var. capitata* [22], *B. juncea* L. [23], and *B. oleracea* L. *var. italic* [24]. Furthermore, metabolic profiling is commonly defined as the identification and quantification of low-molecular-weight metabolites and intermediates produced in many biosynthetic and catabolic pathways in living organisms [25].

**Figure 1:**
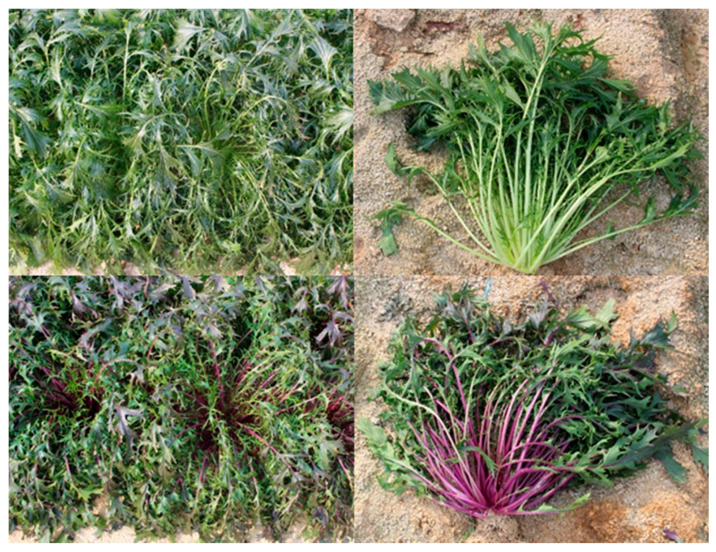
Phenotypes of the green and red cultivars of mizuna.

In this study, green and red mizuna cultivars were used as plant materials (Figure 1). We analyzed the transcriptome and metabolome of these two cultivars using the Illumina Solexa platform, GC-TOFMS, liquid chromatography electrospray ionization tandem mass spectrometric (LC-ESI-MS), and high-performance liquid chromatography (HPLC) and comprehensively described the relationship between all identified metabolites. To our knowledge, this is the first study to provide information on metabolic differences between green and red mizuna through transcriptome and metabolome analyses.

## 2. Materials and Methods

### 2.1. Plant Material

Asia Seed Co., Ltd. (Seoul, Korea) provided plant samples of green and red cultivars of mizuna (*B. rapa* L. var. *japonica*) cultivated at an experimental field of the Asia Seed R&D Center (Icheon, Gyeonggi-do, Korea). Mizuna plants (Figure 1) were harvested after four months, treated with liquid nitrogen (−196 °C), and then stored at −80 °C. Both samples were ground into fine powders for RNA extraction and HPLC analysis.

### 2.2. RNA Extraction and Illumina Sequencing

RNA extraction and Illumina sequencing were performed according to our previously reported method (Jeon et al. [26]). Total RNA was extracted using the Plant Total RNA Mini Kit. The extracted RNA was quantitated using a Nanodrop, and its integrity was measured through denaturing 1% agarose gel electrophoresis. Subsequently, mRNA was separated and purified using Sera-Mag Magnetic Oligo (dT) beads, and cDNA was synthesized for Illumina sequencing using the Illumina/Solexa HiSeq2000 platform. RNA-sequencing analysis was performed to comprehensively compare the characteristics of red and green mizuna transcriptomes. In total, 81,330,120 and 46,286,878 reads were obtained for green and red mizuna, making up 6.18 Gb (giga-bases) and 3.52 Gb, respectively, with an average length of 76 nucleotides per read. After filtering, 61,035,862 and 36,350,466 clean reads were obtained for the green and red mizuna samples, respectively, with Q30 values of 89.69% and 92.31%, respectively, (Table S1). The Bowtie2 software and the DESeq library were used to estimate the expression abundance.

### 2.3. Assembly and Functional Annotation

After quality trimming of the raw RNA-sequencing reads, 81,330,120 and 46,286,878 high-quality clean reads of green and red cultivars of mizuna, respectively, were prepared for transcriptome assembly. The Trinity de novo assembly program (https://github.com/trinityrnaseq/trinityrnaseq/wiki) was used to combine the overlapping reads into contigs without gaps. Furthermore, the filtered reads were aligned back to the de novo assembled transcriptome sequences to validate the assembly and then mapped into the *B. rapa* reference genome from the Brassica database (BRAD) (http://brassicadb.org/brad) using TopHat2 (http://tophat.cbcb.umd.edu) Martin [27] and bowtie2 (http://bowtie-bio.sourceforge.net) (Kim et al. [28]). After normalizing expression levels by using the DESeq package in R, the transcript expression levels were calculated using the Fragments Per Kilobase of transcript per Million mapped reads (FPKM), and a false discovery rate (FDR) was applied to determine the significance cutoff of 0.05 via DESeq. Gene ontology (GO) was performed for the differentially expressed unigenes (DEGs) with an FDR < 0.05 in both cultivars. The Kyoto Encyclopedia of Genes and Genomes (KEGG) and Plant Transcription Factor (PlantTFDB) databases were used for the further analysis of genome networks. The statistical significance of DEGs was determined using the threshold setting (log2-fold changes of ≤−1 or ≥1 and an adjusted *p*-value of ≤0.05).

### 2.4. HPLC Analysis of Desulfo-Glucosinolates

Desulfoglucosinolates were extracted and analyzed as per previously reported procedures by Park et al. [29]. Briefly, 100 mg dried green and red mizuna cultivars were extracted with 1.5 mL of 70 °C boiling aquatic methanol 70% (*v*/*v*) for 5 min and then centrifuged at 11,000× *g* at 4 °C for 15 min. The supernatants were transferred into fresh tubes. The remaining pellets were re-extracted twice more in the same manner. The final collected supernatants were loaded onto a mini-column filled with DEAE-Sephadex A-25, followed by desulfation using 75 μL of an aryl sulfatase solution for 12 h. The resulting desulfoglucosinolate extract was further eluted with 0.5 mL of HPLC-grade water. An Agilent Technologies 1200 Series HPLC System (Palo Alto, CA, USA) equipped with an Inertsil^®^ ODS-3 column (150 × 3.0 mm i.d., particle size 3 μm; GL Sciences, Tokyo, Japan) and an Inertsil^®^ ODS-2 guard column (10 × 2.0 mm i.d., particle size 5 μm) was used to separate glucosinolate components. The HPLC conditions were set as follows: detection wavelength, 227 nm; oven temperature, 40 °C; flow rate, 0.2 mL min^−1^; injection volume, 20 μL. The gradient program used was as follows: solvent A, HPLC-grade water; solvent B, HPLC-grade acetonitrile; 0–18 min, 7–24% B; 18–32 min, 24% B; 32.01 min, rapid drop to 7% B; and 32.01–40 min, 7% B (total 40 min). Quantification of desulfoglucosinolates was performed as previously described by Park et al. [29]. Each compound was quantified using sinigrin (internal standard) and the response factor of individual desulfoglucosinolates relative to the internal standard.

### 2.5. HPLC Analysis of the Phenolic Compounds

Phenolics were extracted as per the previously reported method by Park et al. [30]. Briefly, 1 mL of aqueous methanol (80% *v*/*v*) was added to a fresh tube containing fine powders (100 mg) of dried green and red mizuna cultivars and vortexed thoroughly for 20 s. The extract was sonicated at temperatures below 25 °C for 60 min and centrifuged at 11, 000× *g* for 15 min. Then, the supernatants were collected into fresh tubes. The remaining sludge was used to re-extract phenolics two more times in the same manner. The collected supernatants were dried using a vacuum concentrator and then dissolved in 0.5 mL of methanol. An NS-4000 HPLC system (Futecs, Daejeon, Korea) with a UV−Vis detector and a C_18_ column (250 × 4.6 mm, 5 μm; RStech, Daejeon, Korea) was used to isolate gallic acid, chlorogenic acid, caffeic acid, catechin, epi-catechin, vanillin, and benzoic acid. The gradient program was used according to our previously reported method [31]. The mobile phase consisted of solvent A, ultrapure water containing 0.2% acetic acid, solvent B, and methanol at a flow rate of 1 mL/min. The HPLC running time was 98 min, the oven temperature was 30 °C, and the detection wavelength was 280 nm. Peak identification was performed by comparison with the retention times of the standard chemicals and spike test, and quantification was carried out according to each calibration curve of eight phenolic and organic compounds (Table S2). The standards used for analysis of the phenolic compounds are gallic acid (≥99%), vanillin (99%), catechin hydrate (≥98%), (−)-catechin (≥98%), benzoic acid (≥99.5%), caffeic acid (≥98%), chlorogenic acid (≥95%), 4-hydroxybenzoic acid (99%) and were purchased from Sigma-Aldrich Co., Ltd. (St. Louis, MO, USA).

### 2.6. HPLC and LC-ESI-MS/MS Analysis of Anthocyanins

Anthocyanins were extracted as per our previously reported method (Park et al. [31]). Anthocyanins were extracted from 100 mg fine powders (100 mg) of dried green and red mizuna cultivars, with 2 mL water/formic acid (95:5 *v*/*v*), followed by gentle sonication for 20 min. After centrifugation at 11,000× *g* for 15 min, the extract was syringe-filtered into an LC vial. Different anthocyanins were separated using an Agilent 1200 series HPLC linked to a 4000 Q-Trap LC-ESI-MS/MS system with a Synergi 4 μm POLAR-RP 80A column (250 × 4.6 mm i.d., particle size 4 µm) combined with a Security Guard Cartridges Kit AQ C18 column. The operating conditions and gradient program were used as in our previously reported method (Park et al. [31]). Different anthocyanin quantifications were performed using a standard calibration curve depicted from the commercial anthocyanin (Cyanidin-3-*O*-glucoside (C3G)). C3G was used as an external standard and each anthocyanin was expressed as milligram per C3G gram dry weight (mg/C3G g dry weight).

### 2.7. GC-TOFMS Analysis of Polar Metabolites

Hydrophilic were extracted as per our previously reported method (Park et al. [29]). Fine powders (50 mg) of green and red mizuna were extracted with 1 mL of a chloroform-water-methanol mixture (1:1:2.5 *v*/*v*/*v*) to which 60 µL of ribitol (0.2 g/L) was added as an internal standard. The extract was shaken at 37 °C and 1200× *g* for 30 min and then centrifuged at 12,000× *g* for 15 min. After the supernatants were evaporated in a SpeedVac vacuum concentrator for 3 h, the extract was derivatized by adding 80 μL of methoxy-amine hydrochloride/pyridine (20 g L^−1^) and shaken at 37 °C and 1200× *g* for 2 h. Thereafter, 80 μL of N-methyl-N-(trimethylsilyl)trifluoroacetamide was added and the resulting mixture was heated at 37 °C for 30 min. After centrifugation, the final extract was transferred to a 2 mL glass vial with a micro insert. The analysis equipment and operation conditions of GC-TOFMS were as described in our previous study (Park et al. [29]). The quantification of the polar metabolite was performed using selected ions, and the Chroma-TOF software was used to locate the peaks. The peak identification of the GC-TOF/MS data was performed by comparing their retention times and mass spectrum with standard compounds, in-house library, and MS library (Nist database). ChromaTOF software was used to identify the hydrophilic compounds in mizuna cultivars. The results were filtered with retention time, signal-to-noise ratio (>5:1), and mass spectral matching (based on a match >700) by using reference compounds and the use of an in-house library. As a result, a total of 43 metabolites were identified (i.e., Metabolomics Standards Initiative (MSI) level 1) Viant et al. [32]. The corresponding retention times and their fragment patterns were agreed with our previous data (Park et al. [29]).

### 2.8. Statistical Analysis of Metabolites

A Student’s *t*-test was performed using the Statistical Analysis System (SAS, system 9.4, 2013; SAS Institute, Inc., Cary, NC, USA). A volcano plot and a hierarchical cluster analysis (HCA) were carried out, and Pearson correlations for 76 metabolites identified in these analyses were determined using MetaboAnalyst 4.0 (http://www.metaboanalyst.ca/) with auto-scaling.

## 3. Results and Discussion

### 3.1. Functional Annotation and Classification of the Green and Red Mizuna Transcriptomes

**Figure 2:**
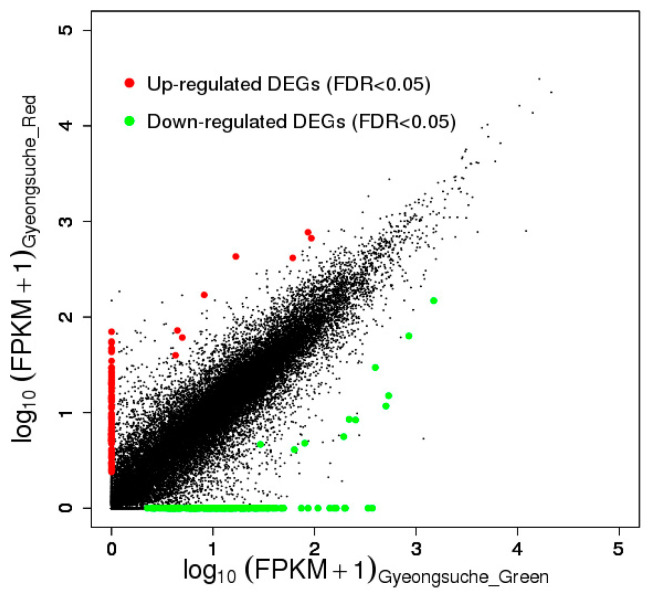
Gene expression levels of green and red mizuna. The differentially expressed genes are shown in red and green, and genes without expression changes are shown in black (false discovery rate (FDR) < 0.05).

The cDNA libraries of the green and red mizuna were mapped to the *B. rapa* reference genome with coverages of 75.5% and 75.4%, respectively, (Table S3). Among the 257 DEGs with an FDR < 0.05, 94 were expressed at higher levels and 163 were expressed at relatively lower levels in the red cultivar than in the green cultivar (Figure 2). A GO analysis of the DEGs was carried out using DAVID (http://david.abcc.ncifcrf.gov/tools.jsp). The DEGs were assigned to the GO terms of biological process (BP) and molecular function (MF) categories as shown in Table 1. In the BP category, the DEGs were assigned to cell death (10 DEGs), apoptosis (eight DEGs), toxin catabolic process (four DEGs), toxin metabolic process (four DEGs), sucrose metabolic process (three DEGs), defense response (19 DEGs), innate immune response (eight DEGs), response to temperature stimulus (nine DEGs), secondary metabolic process (10 DEGs), response to bacterium (seven DEGs), multidrug transport (four DEGs), oxidation reduction (19 DEGs), drug transport (four DEGs), response to drug (four DEGs), and response to reactive oxygen species (five DEGs). In the MF category, the DEGs were assigned to heme binding (11 DEGs), tetrapyrrole binding (11 DEGs), oxygen binding (eight DEGs), iron ion binding (15 DEGs), glutathione transferase activity (four DEGs), electron carrier activity (14 DEGs), and drug transporter activity (four DEGs). This result was consistent with the previous results of Park et al. [33].

### 3.2. Identification of Secondary Metabolite Biosynthetic Genes from the Green and Red Mizuna Transcriptomes

Transcriptome analysis of mizuna cultivars revealed the identification of genes involved in the biosynthesis of secondary metabolites, including glucosinolates, phenolics, and anthocyanins. Based on the Arabidopsis Information Resource (TAIR) website and the basic local alignment search tool (BLAST) program, 12 genes encoding enzymes involved in the glucosinolate biosynthesis pathway and 10 genes encoding enzymes involved in the phenylpropanoid and flavonoid biosynthesis pathways were identified in the transcriptome data of mizuna (Tables S4 and S5). Among these 12 identified genes, the expression of the gene encoding the transcription factor MYB28, which regulates aliphatic glucosinolate biosynthesis, was significantly higher in green mizuna than in red mizuna. In addition, the expression of the structural genes (branched-chain aminotransferase 4 (*BCAT4*); methylthioalkylmalate synthase 1 (*MAM1*); cytochrome p450, family 79, subfamily F, polypeptide 1 (*CYP79F1*); cytochrome P450, family 83, subfamily A, polypeptide 1 (*CYP83A1*); tyrosine transaminase family protein (*SUR1*), 2-oxoglutarate and Fe(II)-dependent oxygenase superfamily protein (*AOP3*), sulfotransferase 17 (*ST5c*), and desulfoglucosinolate sulfotransferase 18 (*ST5b*)), involved in aliphatic glucosinolate biosynthesis were significantly higher in green mizuna. In addition, the expression of the bile acid transporter 5 (*BAT5*) gene, required for aliphatic glucosinolate biosynthesis, was upregulated in green mizuna. In contrast, the expression of the transcription factor MYB34, which regulates indolic glucosinolate biosynthesis, was significantly upregulated in red mizuna (Table S4). Furthermore, the key genes involved in flavonoid biosynthesis (dihydroflavonol 4-reductase (*DFR*), leucoanthocyanidin dioxygenase (*ANS*), UDP-glucose: flavonoid 3-O-glucosyltransferase (*UF3GT*), anthocyanin 5-O-glucosyltransferase (*5GT*), and transparent testa 19 (*TT19*)) as well as several phenylpropanoid biosynthetic genes (cinnamate-4-hydroxylase (*C4H*) and hydroxycinnamoyl-CoA shikimate/quinate hydroxycinnamoyl transferase (*HCT*)) were expressed at higher levels in red mizuna than in green mizuna. In contrast, the 4-coumarate:CoA ligase 5 (*4CL5*) and caffeate O-methyltransferase 1 (*COMT1*) genes, which are involved in the phenylpropanoid pathway, were expressed at higher levels in green mizuna than in red mizuna (Table S5).

### 3.3. Quantification of Glucosinolates in Green and Red Mizuna

Fourteen glucosinolates were detected in green mizuna, whereas only nine glucosinolates were found in red mizuna (Table 2). The level of total glucosinolates was significantly higher in green mizuna than in red mizuna. Specifically, glucoraphanin, glucoalyssin, and gluconapoleiferin were detected only in green mizuna, and progoitrin and 4-hydroxyglucobrassicin levels were significantly higher in green than in red mizuna. In contrast, gluconasturtiin was detected only in red mizuna and glucobrassicin, 4-methoxyglucobrassicin, and neoglucobrassicin were significantly higher in red than in green mizuna. In particular, the highest accumulation patterns of most aliphatic glucosinolates (glucoiberin, progoitrin, glucoraphanin, glucoalyssin, gluconapoleiferin, and gluconapin) were observed in the green cultivar, whereas the red cultivar showed higher production patterns of indolic glucosinolates, represented mainly by glucobrassicin, 4-methoxyglucobrassicin, and neoglucobrassicin. These results were consistent with the results of the DEG analysis, revealing that the expression of the regulatory gene *MYB28* and structural genes (*BCAT4*, *MAM1*, *CYP79F1*, *CYP83A1*, *SUR1*, *AOP3*, *ST5c*, and *ST5b*) responsible for aliphatic glucosinolate biosynthesis was higher in the green than in red mizuna, but revealed that the expression of a regulatory gene *MYB34* involved in indolic glucosinolate biosynthesis was significantly higher in red than in green mizuna. Furthermore, these findings were supported by previous studies reporting that *BoaMYB28* overexpressing lines of Chinese kale showed increased transcript levels of the aliphatic glucosinolate biosynthesis genes and increased levels of aliphatic glucosinolates. In contrast, the transcript levels and aliphatic glucosinolate levels decreased in the *BoaMYB28* RNAi transgenic lines of Chinese kale [34]. Similarly, Frerigmann and Gigolashvilli [35] reported that MYB34 is the main transcription factor regulating indolic glucosinolate biosynthesis.

### 3.4. Quantification of Phenolic and Organic Compounds in Green and Red Mizuna

Five phenolic and two organic compounds related to the biosynthesis of phenolic compounds were detected in both green and red mizuna cultivars using HPLC (Table 3), whereas a total of 12 anthocyanins were identified only in red mizuna (Table 4). For the individual compounds, the levels of caffeic acid, (−)-epicatechin, and vanillin were higher in green mizuna, whereas those of gallic acid and catechin were higher in red mizuna. Furthermore, cyanidins were the major anthocyanins ubiquitous in red mizuna, and the red color might be derived from the identified cyanidin derivatives in the red cultivar [36]. These HPLC results were in accordance with the results of the analysis of DEGs which revealed that the expression of structural genes (*C4H*, *DFR*, *ANS*, *UF3GT*, *5GT*, and *TT19*) responsible for the phenylpropanoid and flavonoid biosynthesis was significantly higher in red mizuna. Similarly, Jeon et al. [26] reported that higher levels of cyanidin derivatives were present in red kale than in green kale, which is consistent with the higher expression of anthocyanin biosynthetic genes in red kale via metabolome and transcriptome analysis.

### 3.5. Quantification of Phenolic and Organic Compounds in Green and Red Mizuna

A total of 43 hydrophilic (two phenolic acids, three photorespiration intermediates, four TCA cycle intermediates, five organic acids, 18 amino acids, and 11 sugars) were identified and quantified in both cultivars using GC-TOFMS (Table S6). A comparison of amino acid levels between the green and red mizuna cultivars indicated that the levels of valine, serine, leucine, isoleucine, proline, glycine, threonine, β-alanine, aspartic acid, methionine, pyroglutamic acid, asparagine, glutamine, phenylalanine, tryptophan, and glutamic acid were significantly higher in green mizuna, whereas 4-aminobutyric acid levels were only higher in red mizuna. In particular, the glutamine content was consistent with the transcript levels of glutamine synthetase 1;4 (Table S7). Among the identified sugars, xylose, fructose, glucose, mannose, and glycerol levels were significantly higher in green mizuna. However, the levels of sucrose, maltose, trehalose, raffinose, and inositol were higher in red mizuna (Figure S1). Sucrose synthesis involves a two-step process catalyzed by two different enzymes, sucrose-6-phosphate synthase (SPS) and sucrose-6-phosphate phosphatase (SPP), in plants. In the first step of the sucrose biosynthetic pathway catalyzed by the SPS, sucrose-6-phosphate is synthesized from uracil-diphosphate glucose (UDP-glucose) and fructose-6-phosphate through the SPS activity. Next, SPP rapidly dephosphorylates sucrose-6-phosphate to sucrose and inorganic phosphate [37]. The produced sucrose can be further degraded to fructose and UDP-glucose by the activity of sucrose synthase [38]. In this study, the sucrose level was consistent with the analysis of DEGs indicating the higher expression of sucrose-phosphate synthase family protein (SPS4F) and sucrose-phosphatase 1 (SPP1) and lower expression of sucrose synthase 3 (SUS3) in red mizuna.

**Figure 3:**
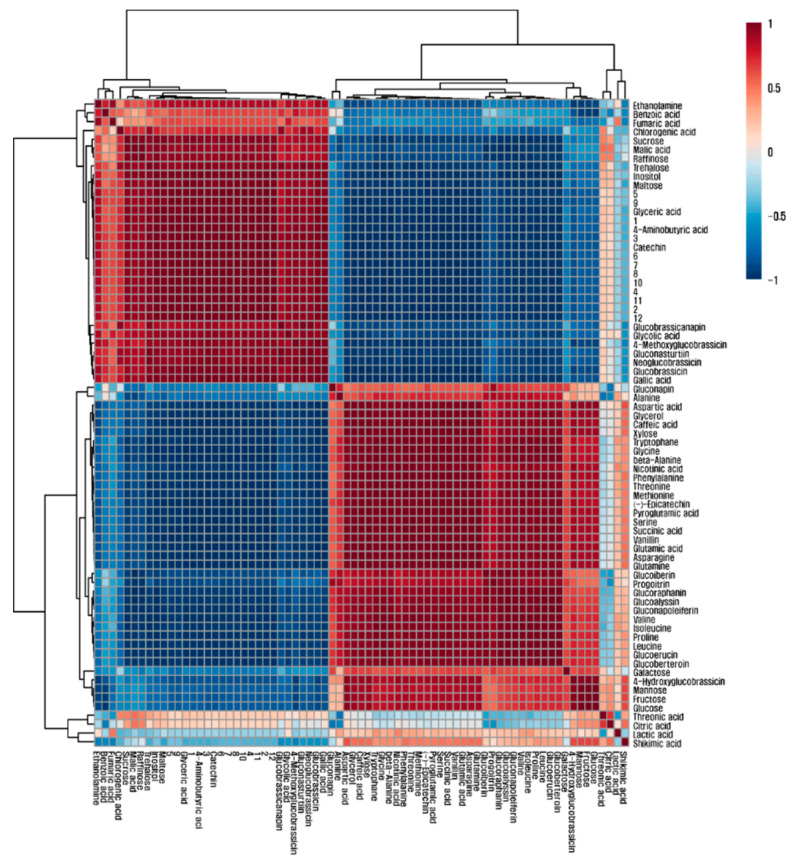
Correlation matrix of metabolites from green and red mizuna cultivars. Each square indicates the Pearson’s correlation coefficient of a pair of compounds, and the value of the correlation coefficient is represented by the intensity of the color ranging from deep blue to deep red, as indicated on the color scale. 1, Cyanidin 3-diglucoside-5-glucoside; 2, Cyanidin 3-(sinapoyl)diglucoside-5-glucoside; 3, Cyanidin 3-(caffeoyl)(p-coumaroyl)diglucoside-5-glucoside; 4, Cyanidin 3-(glycolpyranosyl-sinapoyl)diglucoside-5-glucoside; 5, Cyanidin 3-(p-coumaroyl)(sinapoyl)triglucoside-5-glucoside; 6, Cyanidin 3-(sinapoyl)glucoside-5-glucoside; 7, Cyanidin 3-(p-coumaroyl)diglucoside-5-glucoside; 8, Cyanidin 3-(sinapoyl)diglucoside-5-glucoside; 9, Cyanidin 3-(p-coumaroyl)(sinapoyl)diglucoside-5-glucoside; 10, Cyanidin 3-(feruloyl)(sinapoyl)diglucoside-5-glucoside; 11, Cyanidin 3-(sinapoyl)(sinapoyl)diglucoside-5-glucoside; 12, Cyanidin 3-(feruloyl)(sinapoyl)diglucoside-5-glucoside.

To obtain an insight into the correlation among the 73 metabolites identified in green and red mizuna cultivars, an HCA was performed on the datasets using Pearson’s correlation results (Figure 3). Glutamine, glutamic acid, aspartic acid, and asparagine comprise the metabolic network involved in nitrogen metabolism into amino acids. In this study, glutamic acid was highly positively correlated with glutamine (*r* = 0.99958, *p* < 0.0001), aspartic acid (*r* = 0.98871, *p* < 0.0005), and asparagine (*r* = 0.99881, *p* < 0.0001). Strong correlations were observed between glutamic acid and the amino acids of the glutamate family, including pyroglutamic acid (*r* = 0.99872, *p* < 0.0001) and proline (*r* = 0.96723, *p* < 0.005), as well as between aspartic acid and the amino acids of the aspartate family, including asparagine (*r* = 0.98857, *p* < 0.005), threonine (*r* = 0.97286, *p* < 0.005), methionine (*r* = 0.97009, *p* < 0.01), isoleucine (*r* = 0.89007, *p* < 0.05), and beta-alanine (*r* = 0.96221, *p* < 0.005). In particular, sucrose had a strong positive correlation with 12 anthocyanins identified (*r* > 0.9, *p* < 0.0001).

Sucrose is one of the main regulators of plant growth processes [39] and acts as an energy source and an intermediate for metabolic processes [36]. The correlations between sucrose and 12 anthocyanins identified revealed a positive effect of sucrose on anthocyanin production, as supported by previous studies reporting the effect of sucrose on anthocyanin biosynthesis. Shin et al. reported that the calcium-dependent sucrose uptake, activated by the external sucrose treatment, increased the endogenous sugar pools, which induced anthocyanin accumulation by activating anthocyanin biosynthetic regulatory genes (production of anthocyanin pigment 1 (*PAP1*) and *PAP2*) and structural (*CHS*, *DFR*, *ANS*, and *UF3GT*) genes [40]. Additionally, the *pho3* mutant showed large pools of sugars (starch, fructose, sucrose, and glucose) and increased anthocyanin production [41], and the endogenous sucrose pool had a strong positive correlation with anthocyanin in mulberry fruits [42]. The exogenous sucrose supply enhanced the levels of pelargonidin derivatives in postharvest strawberry fruits [43] and anthocyanin production in grapevine cell cultures [44].

## 4. Conclusions

To our knowledge, this is the first study to provide a comprehensive transcriptome and metabolome analysis of primary and secondary metabolites in green and red mizuna. Based on the high-throughput transcriptome data, the primary metabolite biosynthesis genes, including three genes related to sucrose metabolism and one gene related to glutamine metabolism, as well as secondary metabolite biosynthesis genes, include 12 genes involved in glucosinolate biosynthesis and 10 genes responsible for phenylpropanoid and flavonoid biosynthesis in mizuna. Furthermore, 14 glucosinolates, 12 anthocyanins, five phenolics, two organic acids, and 43 hydrophilic were detected in red and green mizuna cultivars using the HPLC, ESI-LC/MS/MS, and GC-TOFMS analyses. Through a comparative transcriptome and metabolome analysis, this study showed that the green mizuna contained a higher content of aliphatic glucosinolates in accordance with the expression of genes involved in aliphatic glucosinolate biosynthesis. In contrast, the red cultivar had a higher content of indolic glucosinolates and anthocyanin, consistent with the expression of genes responsible for indolic glucosinolate and flavonoid biosynthesis. Furthermore, a strong positive correlation between sucrose and anthocyanins was observed to support the positive effect of sucrose on anthocyanin biosynthesis. Taken together, these findings may help to develop breeding strategies and also to improve the biosynthesis of glucosinolates and anthocyanins in mizuna.

## Figures and Tables

**Table 1:** Gene ontology terms (*p*-value and fold enrichment) that were significant in the enrichment analysis in the gene list from the red vs. green mizuna cultivar comparison.

Category	Term	Count	*p*-Value	Fold Enrichment
Biological process	GO:0008219~cell death	10	0.00092	4
	GO:0006915~apoptosis	8	0.0016	4.7
	GO:0009407~toxin catabolic process	4	0.0104	8.8
	GO:0009404~toxin metabolic process	4	0.0104	8.8
	GO:0005985~sucrose metabolic process	3	0.0119	17.8
	GO:0006952~defense response	19	0.0125	1.9
	GO:0045087~innate immune response	8	0.0192	2.9
	GO:0009266~response to temperature stimulus	9	0.024	2.6
	GO:0019748~secondary metabolic process	10	0.0242	2.4
	GO:0009617~response to bacterium	7	0.0339	2.9
	GO:0006855~multidrug transport	4	0.0379	5.4
	GO:0055114~oxidation reduction	19	0.0434	1.6
	GO:0015893~drug transport	4	0.0445	5
	GO:0042493~response to drug	4	0.0459	5
	GO:0000302~response to reactive oxygen species	5	0.0497	3.6
	GO:0020037~heme binding	11	0.00382	3.0
	GO:0046906~tetrapyrrole binding	11	0.00653	2.8
	GO:0019825~oxygen binding	8	0.00818	3.5
Molecular function	GO:0005506~iron ion binding	15	0.01239	2.1
	GO:0004364~glutathione transferase activity	4	0.01243	8.2
	GO:0009055~electron carrier activity	14	0.01295	2.1
	GO:0015238~drug transporter activity	4	0.04875	4.9

**Table 2:** Glucosinolate content in green and red kale (µmol/g dry weight).

Class	Name	Green Mizuna	Red Mizuna
	Glucoiberin	0.14 ± 0.03 ^1^	0.07 ± 0.00
	Progoitrin	0.31 ± 0.05 *^,2^	0.16 ± 0.04
	Glucoraphanin	0.14 ± 0.04	N.D. ^3^
	Glucoalyssin	0.32 ± 0.08	N.D.
Aliphatic glucosinolate	Gluconapoleiferin	0.22 ± 0.05	N.D.
	Gluconapin	6.45 ± 1.09	4.67 ± 1.63
	Glucobrassicanapin	0.40 ± 0.07	2.37 ± 0.84
	Glucoerucin	3.45 ± 0.59	N.D.
	Glucoberteroin	2.71 ± 0.47	N.D.
Phenethyl glucosinolate	Gluconasturtiin	N.D.	0.16 ± 0.04
Indolic glucosinolate	4-Hydroxyglucobrassicin	0.26 ± 0.11 *	0.05 ± 0.02
	Glucobrassicin	0.84 ± 0.09	1.43 ± 0.03 ***
	4-Methoxyglucobrassicin	0.48 ± 0.05	0.70 ± 0.03 **
	Neoglucobrassicin	0.08 ± 0.01	0.28 ± 0.05 **
	Total	15.81 ± 2.51 *	9.88 ± 2.66

^1^ The values represent the means ± standard deviation of three biological replicates. ^2^ Asterisks indicate significant differences as determined by the Student’s *t*-test (* *p* < 0.05; ** *p* < 0.01, *** *p* < 0.001). ^3^ N.D., Not detected.

**Table 3:** Phenolic and organic compounds in green and red mizuna (µm/g dry weight).

Class	Name	Green Mizuna	Red Mizuna
Phenolic acid	Gallic acid	1.48 ± 0.18	4.71 ± 0.25 *** ^1^
	Chlorogenic acid	176.51 ± 2.88	184.86 ± 5.56
	Caffeic acid	120.09 ± 6.03 ***	64.82 ± 1.47
Flavonoid	Catechin	154.16 ± 0.90	234.07 ± 6.15 **
	(−)-Epicatechin	1351.16 ± 21.66 ***	847.38 ± 42.12
Organic compound	Vanillin	64.71 ± 3.82 ***	5.09 ± 1.28
	Benzoic acid	195.20 ± 22.33	220.89 ± 14.38
	Total	2063.30 ± 23.08 ***	1561.82 ± 66.89

^1^ The values represent the means ± standard deviation of three biological replicates. Asterisks indicate significant differences as determined by the Student’s *t*-test (** *p* < 0.01, *** *p* < 0.001).

**Table 4:** Anthocyanin content in green and red kale. (µmol/g dry weight).

Class	Name	Green Mizuna	Red Mizuna
Flavonoids	Cyanidin 3-diglucoside-5-glucoside	N.D. ^1^	0.02 ± 0.00 ^2^
	Cyanidin 3-(sinapoyl)diglucoside-5-glucoside	N.D.	0.16 ± 0.01
	Cyanidin 3-(caffeoyl)(*p*-coumaroyl)diglucoside-5-glucoside	N.D.	0.03 ± 0.00
	Cyanidin 3-(glycopyranosyl-sinapoyl)diglucoside-5-glucoside	N.D.	0.13 ± 0.01
	Cyanidin 3-(*p*-coumaroyl)(sinapoyl)triglucoside-5-glucoside	N.D.	0.02 ± 0.00
	Cyanidin 3-(sinapoyl)glucoside-5-glucoside	N.D.	0.20 ± 0.02
	Cyanidin 3-(*p*-coumaroyl)diglucoside-5-glucoside	N.D.	0.11 ± 0.01
	Cyanidin 3-(sinapoyl)diglucoside-5-glucoside	N.D.	0.25 ± 0.02
	Cyanidin 3-(*p*-coumaroyl)(sinapoyl)diglucoside-5-glucoside	N.D.	0.04 ± 0.01
	Cyanidin 3-(feruloyl)(sinapoyl)diglucoside-5-glucoside	N.D.	0.35 ± 0.02
	Cyanidin 3-(sinapoyl)(sinapoyl)diglucoside-5-glucoside	N.D.	0.76 ± 0.04
	Cyanidin 3-(feruloyl)(sinapoyl)diglucoside-5-glucoside	N.D.	0.15 ± 0.01
	Total		2.49 ± 0.18

^1^ N.D., Not detected. ^2^ The values represent the means ± standard deviation of three biological replicates.

## Supplementary Materials

The following are available online at https://www.mdpi.com/2304-8158/9/8/1079/s1. Figure S1: Volcano plot of differentially accumulated metabolites between green and red mizuna. The y-axis is the negative log10 of *p* values and the x-axis is log2 of fold change. Red indicates that the metabolites identified in green mizuna were significantly higher than those of red mizuna, blue indicates that the metabolites identified in green mizuna were significantly lower than those of red mizuna, and grey indicates metabolites with no significant difference. Table S1: Summary of RNA sequence data, Table S2: Validation of HPLC analysis of phenolic compounds, Table S3: Summary of genome mapping, Table S4: Putative glucosinolate biosynthetic genes in the mizuna transcriptome., Table S5: Putative phenylpropanoid and anthocyanin biosynthetic genes in the mizuna transcriptome., Table S6: Identified metabolites in GC-TOFMS chromatograms from mizuna extract., Table S7: Putative sucrose and glutamine biosynthetic genes in the mizuna transcriptome., Table S8. The fold changes of the red relative to the green mizuna corresponding to Figure S1.

## References

[1] Bjorkman M., Klingen I., Birch A.N.E., Bones A.M., Bruce T.J.A., Johansen T.J., Meadow R., Molmann J., Seljasen R., Smart L.E. (2011). Phytochemicals of Brassicaceae in plant protection and human health—Influences of climate, environment and agronomic practice. Phytochemistry.

[2] Cardone M., Mazzoncini M., Menini S., Rocco V., Senatore A., Seggiani M., Vitolo S. (2003). Brassica carinata as an alternative oil crop for the production of biodiesel in Italy: Agronomic evaluation, fuel production by transesterification and characterization. Biomass Bioenergy.

[3] Dominguez-Perles R., Mena P., Garcia-Viguera C., Moreno D.A. (2014). Brassica Foods as a Dietary Source of Vitamin C: A Review. Crit. Rev. Food Sci..

[4] Jahangir M., Kim H.K., Choi Y.H., Verpoorte R. (2009). Health-Affecting Compounds in Brassicaceae. Compr. Rev. Food Sci. Food Saf..

[5] Prakash D., Gupta C. (2012). The phytochemicals of nutraceutical importance. J. Complement Integr. Med..

[6] Sun J.H., Zhang M.L., Chen P. (2016). GLS-Finder: A Platform for Fast Profiling of Glucosinolates in Brassica Vegetables. J. Agric. Food Chem..

[7] Herr I., Buchler M.W. (2010). Dietary constituents of broccoli and other cruciferous vegetables: Implications for prevention and therapy of cancer. Cancer Treat. Rev..

[8] Kim Y.B., Li X., Kim S.J., Kim H.H., Lee J., Kim H., Park S.U. (2013). MYB Transcription Factors Regulate Glucosinolate Biosynthesis in Different Organs of Chinese Cabbage (Brassica rapa ssp pekinensis). Molecules.

[9] Douglas C.J. (1996). Phenylpropanoid metabolism and lignin biosynthesis: From weeds to trees. Trends Plant Sci..

[10] Higdon J.V., Delage B., Williams D.E., Dashwood R.H. (2007). Cruciferous vegetables and human cancer risk: Epidemiologic evidence and mechanistic basis. Pharm. Res..

[11] Cartea M.E., Francisco M., Soengas P., Velasco P. (2011). Phenolic Compounds in Brassica Vegetables. Molecules.

[12] Treutter D. (2006). Significance of flavonoids in plant resistance: A review. Environ. Chem. Lett..

[13] Hoch W.A., Zeldin E.L., McCown B.H. (2001). Physiological significance of anthocyanins during autumnal leaf senescence. Tree Physiol..

[14] Chalker-Scott L. (1999). Environmental significance of anthocyanins in plant stress responses. Photochem. Photobiol..

[15] Nystrand O., Granstrom A. (1997). Post-dispersal predation on Pinus sylvestris seeds by Fringilla spp: Ground substrate affects selection for seed color. Oecologia.

[16] Wrolstad R.E. (2004). Interaction of natural colors with other ingredients—Anthocyanin pigments—Bioactivity and coloring properties. J. Food Sci..

[17] Kalisz A., Sekara A., Kostrzewa J. (2012). Effect of growing date and cultivar on the morphological parameters and yield of Brassica rapa var. japonica. Acta Sci. Pol. Hortorum Cultus.

[18] Khanam U.K.S., Oba S., Yanase E., Murakami Y. (2012). Phenolic acids, flavonoids and total antioxidant capacity of selected leafy vegetables. J. Funct. Foods.

[19] Kim O.K., Murakami A., Nakamura Y., Ohigashi H. (1998). Screening of edible Japanese plants for nitric oxide generation inhibitory activities in RAW 264.7 cells. Cancer Lett..

[20] Li D.J., Deng Z., Qin B., Liu X.H., Men Z.H. (2012). De novo assembly and characterization of bark transcriptome using Illumina sequencing and development of EST-SSR markers in rubber tree (Hevea brasiliensis Muell. Arg.). BMC Genom..

[21] Tong C.B., Wang X.W., Yu J.Y., Wu J., Li W.S., Huang J.Y., Dong C.H., Hua W., Liu S.Y. (2013). Comprehensive analysis of RNA-seq data reveals the complexity of the transcriptome in Brassica rapa. BMC Genom..

[22] Kim H.A., Lim C.J., Kim S., Choe J.K., Jo S.H., Baek N., Kwon S.Y. (2014). High-Throughput Sequencing and De Novo Assembly of *Brassica oleracea* var. *Capitata*, L. for Transcriptome Analysis. PLoS ONE.

[23] Sinha S., Raxwal V.K., Joshi B., Jagannath A., Katiyar-Agarwal S., Goel S., Kumar A., Agarwal M. (2015). De novo transcriptome profiling of cold-stressed siliques during pod filling stages in Indian mustard (*Brassica juncea* L.). Front. Plant Sci..

[24] Gao J.J., Yu X.X., Ma F.M., Li J. (2014). RNA-Seq Analysis of Transcriptome and Glucosinolate Metabolism in Seeds and Sprouts of Broccoli (*Brassica oleracea* var. *italic*). PLoS ONE.

[25] Park C.H., Morgan A.M.A., Park B.B., Lee S.Y., Lee S., Kim J.K., Park S.U. (2019). Metabolic Analysis of Four Cultivars of Liriope platyphylla. Metabolites.

[26] Jeon J., Kim J.K., Kim H., Kim Y.J., Park Y.J., Kim S.J., Kim C., Park S.U. (2018). Transcriptome analysis and metabolic profiling of green and red kale (*Brassica oleracea* var. *acephala*) seedlings. Food Chem..

[27] Martin M. (2011). Cutadapt removes adapter sequences from high-throughput sequencing reads. EMBnet J..

[28] Kim D., Pertea G., Trapnell C., Pimentel H., Kelley R., Salzberg S.L. (2013). TopHat2: Accurate alignment of transcriptomes in the presence of insertions, deletions and gene fusions. Genome Biol..

[29] Park C.H., Yeo H.J., Park S.Y., Kim J.K., Park S.U. (2019). Comparative Phytochemical Analyses and Metabolic Profiling of Different Phenotypes of Chinese Cabbage (Brassica Rapa ssp. Pekinensis). Foods.

[30] Park C.H., Kim N.S., Park J.S., Lee S.Y., Lee J.W., Park S.U. (2019). Effects of Light-Emitting Diodes on the Accumulation of Glucosinolates and Phenolic Compounds in Sprouting Canola (*Brassica napus* L.). Foods.

[31] Park C.H., Yeo H.J., Kim N.S., Eun P.Y., Kim S.J., Arasu M.V., Al-Dhabi N.A., Park S.Y., Kim J.K., Park S.U. (2017). Metabolic profiling of pale green and purple kohlrabi (*Brassica oleracea* var. *gongylodes*). Appl. Biol. Chem..

[32] Viant M.R., Kurland I.J., Jones M.R., Dunn W.B. (2017). How close are we to complete annotation of metabolomes?. Curr. Opin. Chem. Biol..

[33] Park C.H., Yeo H.J., Park Y.E., Baek S.A., Kim J.K., Park S.U. (2019). Transcriptome Analysis and Metabolic Profiling of Lycoris Radiata. Biology.

[34] Yin L., Chen H.C., Cao B., Lei J.J., Chen G.J. (2017). Molecular Characterization of MYB28 Involved in Aliphatic Glucosinolate Biosynthesis in Chinese Kale (*Brassica oleracea* var. *alboglabra* Bailey). Front. Plant Sci..

[35] Frerigmann H., Gigolashvili T. (2014). MYB34, MYB51, and MYB122 Distinctly Regulate Indolic Glucosinolate Biosynthesis in Arabidopsis thaliana. Mol. Plant.

[36] Park C.H., Yeo H.J., Kim N.S., Park Y.E., Park S.Y., Kim J.K., Park S.U. (2018). Metabolomic Profiling of the White, Violet, and Red Flowers of Rhododendron schlippenbachii Maxim. Molecules.

[37] Maloney V.J., Park J.Y., Unda F., Mansfield S.D. (2015). Sucrose phosphate synthase and sucrose phosphate phosphatase interact in planta and promote plant growth and biomass accumulation. J. Exp. Bot..

[38] Babb V.M., Haigler C.H. (2001). Sucrose phosphate synthase activity rises in correlation with high-rate cellulose synthesis in three heterotrophic systems. Plant Physiol..

[39] Lastdrager J., Hanson J., Smeekens S. (2014). Sugar signals and the control of plant growth and development. J. Exp. Bot..

[40] Shin D.H., Choi M.G., Lee H.K., Cho M., Choi S.B., Choi G., Park Y.I. (2013). Calcium dependent sucrose uptake links sugar signaling to anthocyanin biosynthesis in Arabidopsis. Biochem. Bioph. Res. Commun..

[41] Zakhleniuk O.V., Raines C.A., Lloyd J.C. (2001). pho3: A phosphorus-deficient mutant of *Arabidopsis thaliana* (L.) Heynh. Planta.

[42] Zhao S.C., Park C.H., Yang J.L., Yeo H.J., Kim T.J., Kim J.K., Park S.U. (2019). Molecular characterization of anthocyanin and betulinic acid biosynthesis in red and white mulberry fruits using high-throughput sequencing. Food Chem..

[43] Li D., Zhang X.C., Xu Y.Q., Li L., Aghdam M.S., Luo Z.S. (2019). Effect of exogenous sucrose on anthocyanin synthesis in postharvest strawberry fruit. Food Chem..

[44] Ferri M., Righetti L., Tassoni A. (2011). Increasing sucrose concentrations promote phenylpropanoid biosynthesis in grapevine cell cultures. J. Plant Physiol..

