# Supplementary material for: Transcriptome Analysis and Metabolic Profiling of Green and Red Mizuna (Brassica rapa L. var. japonica)
Source: Foods. 2020 Aug 8;9(8):1079. doi: 10.3390/foods9081079 (PMC7466343; doi:10.3390/foods9081079)
Supplement: Supplementary file 1 [file foods-09-01079-s001.pdf]

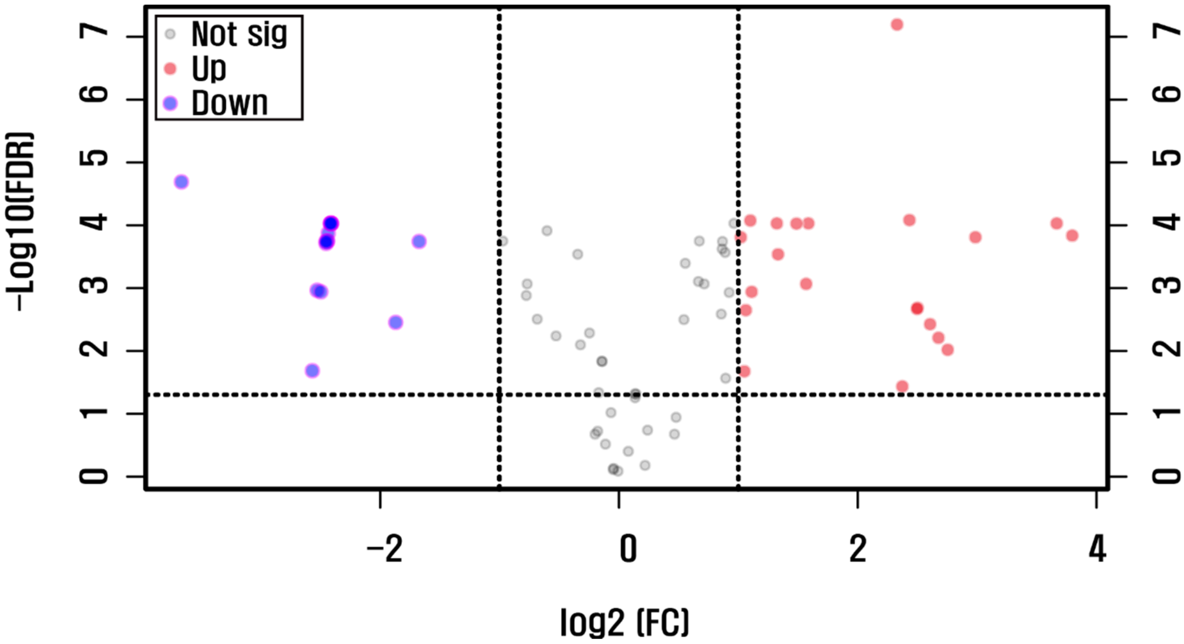

Figure S1. Volcano plot of differentially accumulated metabolites between green and red mizuna.

Table S1. Summary of RNA sequence data.

	Green Mizuna	Red Mizuna
Total reads	81 330 120	46 286 878
Mean length (bp)	76	76
Total base pairs	6 181 089 120	3 517 802 728
Total Bases(Gb)	6.18	3.52
Q30_ More Bases (%)	89.69%	92.31%
Clean reads	61 035 862	36 350 466

Table S2. Validation of HPLC analysis of phenolic compounds.

Compound	Linear Regression Equation	R^2	LOD (ppm)	LOQ (ppm)	Recovery (%)
Gallic acid	y = 32.8959x − 26.1737	0.9999	3.05	9.24	96.57 ± 2.51
Chlorogenic acid	y = 17.795x - 70.351	0.9999	13.05	39.54	99.01 ± 2.47
Caffeic acid	y = 39.983x - 65.708	0.9999	16.21	49.13	102.14 ± 3.67
Catechin	$y = 7.8897x - 40.2424$	0.9999	15.04	45.58	104.09 ± 11.25
(-)-Epicatechin	y = 8.5989x − 8.3356	0.9999	13.80	41.82	100.28 ± 0.80
Vanillin	y = 48.0953 x + 156.7032	0.9998	12.09	36.63	94.28 ± 11.96
Benzoic acid	y = 7.5252x - 37.3870	0.9997	29.76	90.17	102.81 ± 5.32

Table S3. Summary of genome mapping.

Ref. Genome	Sample	Input Read Pairs	Read 1				Read 2				Over-all Map. %	Aligned Pairs	Concord. Pairs %
			Mapped	(%)	Multiply Aligned	(%)	Mapped	(%)	Multiply Aligned	(%)			
Brassica rapa	Gyeongsuche-Green	50 658 466	39 254 352	77.5	11 369 528	29.0	37 257 723	73.5	10 649 355	28.6	75.5	34 539 899	65.3
(BRAD, 285BM)	Gyeongsuche-Red	48 384 885	37 463 805	77.4	21 919 647	58.5	35 531 182	73.4	20 592 356	58.0	75.4	33 356 155	64.8

Table S4. Putative glucosinolate biosynthetic genes in the mizuna transcriptome.

Class	Gene name	Gene ID	TAIR ID	Green Mizuna	Red Mizuna	Fold change[1]	pval
Aliphatic glucosinolate	Myb domain protein 28 (MYB28)	Bra035929	AT5G61420.2	23.9179	5.0997	-2.2296	0.02425
	Branched-chain aminotransferase 4 (BCAT4)	Bra001761	AT3G19710.1	84.3863	29.6737	-1.50782	0.0489
	Branched-chain aminotransferase 4 (BCAT4)	Bra022448	AT3G19710.1	184.82	47.5095	-1.95983	0.00805
	Methylthioalkylmalate synthase 1 (MAM1)	Bra029355	AT5G23010.1	103.382	31.038	-1.73587	0.01655
	Cytochrome p450, family 79, subfamily F, polypeptide 1 (CYP79F1)	Bra026058	AT1G16410.1	182.34	59.5347	-1.61483	0.0276
	Cytochrome P450, family 83, subfamily A, polypeptide 1 (CYP83A1)	Bra032734	AT4G13770.1	238.124	84.9993	-1.48619	0.0418
	Tyrosine transaminase family protein (SUR1)	Bra036703	AT2G20610.1	22.8849	5.58499	-2.03477	0.03975
	2-oxoglutarate (2OG) and Fe(II)-dependent oxygenase superfamily protein (AOP3)	Bra018521	AT4G03050.2	86.4105	31.2526	-1.46723	0.0419
	Sulfotransferase 17 (ST5c)	Bra025668	AT1G18590.1	39.3444	5.28088	-2.89731	0.00645
	Desulfo-glucosinolate sulfotransferase 18 (ST5b)	Bra027880	AT1G74090.1	63.6444	20.4713	-1.63643	0.0412
	Bile acid transporter 5 (BAT5)	Bra029434	AT4G12030.2	27.8445	3.46954	-3.00457	0.0151
Indolic glucosinolate	Myb domain protein 34 (MYB34)	Bra013000	AT5G60890.1	0	1.65278	-	0.0119
	Myb domain protein 34 (MYB34)	Bra029349	AT5G60890.1	0	1.27199	-	0.01525
Benzoic acid-containing glucosinolates	Beta-hydroxyisobutyryl-CoA hydrolase 1 (CHY1)	Bra039968	AT5G65940.1	49.62	1.96501	-4.65831	0.0178

[1] Fold change, log2-fold change of the red mizuna relative to the green Mizuna.

Table S5. Putative phenylpropanoid and anthocyanin biosynthetic genes in the mizuna transcriptome.

Class	Gene name	Gene ID	TAIR ID	Green Mizuna	Red Mizuna	Fold change[1]	pval
Phenylpropanoids	Cinnamate-4-hydroxylase (C4H)	Bra022803	AT2G30490.1	24.4935	71.419	1.54391	0.0445
	4-coumarate:CoA ligase 5 (4CL5)	Bra031265	AT3G21230.1	6.08003	0.482542	-3.65535	0.04465
	Caffeate O-methyltransferase 1 (COMT1)	Bra029041	AT5G54160.1	206.317	32.7281	-2.65626	0.0013
	Hydroxycinnamoyl-CoA shikimate/quinate hydroxycinnamoyl transferase (HCT)	Bra033526	AT5G48930.1	0	5.05747	-	0.0001
Flavonoid	Dihydroflavonol 4-reductase (DFR)	Bra027457	AT5G42800.1	7.17915	169.264	4.55932	0.00015
	Leucoanthocyanidin dioxygenase (ANS)	Bra013652	AT4G22880.2	6.48216	69.4189	3.42078	0.00235
	UDP-glucose:flavonoid 3-O-glucosyltransferase (UF3GT)	Bra035004	AT5G54060.1	0	45.7696	-	5.00E-05
	Anthocyanin 5-O-glucosyltransferase (5GT)	Bra038445	AT4G14090.1	2.43633	83.8857	5.10564	0.0029
	Transparent Testa 19 (TT19)	Bra023602	AT5G17220.1	3.04309	66.7272	4.45467	0.0233
	Transparent Testa 19 (TT19)	Bra008570	AT5G17220.1	0	45.8136	-	5.00E-05
Isoflavonoid	Isoflavone reductase (IFR)	Bra015853	AT1G75280.1	34.3463	7.55466	-2.18472	0.0151

[1] Fold change, log2-fold change of the red Mizuna relative to the green Mizuna.

Table S6. Identified metabolites in GC-TOFMS chromatograms from mizuna extract.

No.	Compound	RT [a]	RRT [b]	Quatification ion [c]
1	lactic acid	4.79	0.441	147
2	valine	5.19	0.478	146
3	alanine	5.29	0.488	116
4	glycolic acid	6.40	0.590	147
5	serine	6.51	0.600	116
6	ethanolamine	7.04	0.649	174
7	glycerol	7.06	0.651	147
8	leucine	7.08	0.652	158
9	isoleucine	7.29	0.672	158
10	proline	7.38	0.680	142
11	nicotinic acid	7.42	0.684	180
12	glycine	7.44	0.686	174
13	succinic acid	7.51	0.692	147
14	glyceric acid	7.61	0.701	147
15	fumaric acid	7.85	0.724	245
16	threonine	8.13	0.749	219
17	β-alanine	8.55	0.788	174
18	malic acid	9.05	0.834	147
19	aspartic acid	9.19	0.847	100
20	methionine	9.36	0.863	176
21	pyroglutamic acid	9.41	0.867	156
22	4-aminobutyric acid	9.43	0.869	174
23	threonic acid	9.58	0.882	147
24	glutamic acid	10.12	0.933	246
25	phenylalanine	10.25	0.945	218
26	xylosse	10.33	0.952	103
27	asparagine	10.52	0.970	116
IS	ribitol (IS)	10.85	1.000	217
28	glutamine	11.30	1.041	156
29	shikimic acid	11.45	1.055	204
30	citric acid	11.57	1.066	273
31	quinic acid	11.82	1.089	345
32	fructose	11.90	1.097	103
33	galactose	12.05	1.111	147
34	glucose	12.09	1.114	147

35	mannose	12.25	1.129	147
36	inositol	13.38	1.233	305
37	ferulic acid	13.49	1.243	338
38	tryptophane	14.23	1.311	202
39	sinapinic acid	14.39	1.326	338
40	sucrose	16.34	1.506	217
41	maltose	16.84	1.552	147
42	trehalose	16.87	1.555	191
43	raffinose	20.06	1.849	217

[a]Retention time (min). [b]Relative retention time (retention time of the analyte/retention time of the internal standard). [c]Specific mass ion used for quantification.

Table S7. Putative sucrose and glutamine biosynthetic genes in the mizuna transcriptome.

Class	Gene name	Gene ID	TAIR ID	Green Mizuna	Red Mizuna	Fold change[1]	pval
	Sucrose synthase 3 (SUS3)	Bra036282	AT4G02280.1	252.55	7.37373	-5.09803	0.00005
Sucrose	Sucrose-phosphate synthase family protein (SPS4F)	Bra033195	AT4G10120.2	3.45811	71.3118	4.36609	0.00005
	Sucrose-phosphatase 1 (SPP1)	Bra014262	AT1G51420.1	0	6.57013	-	0.00005
Amino acid	Glutamine synthetase 1;4 (GLN1;4)	Bra008612	AT5G16570.1	503.896	10.712	-5.55582	0.00005

[1] Fold Change, log2-fold changes of the red relative to the green Mizuna.

Table S8. The fold changes of the red relative to the green mizuna corresponding to Figure S1.

Compound	FC	log2(FC)	p.adjusted	-log10(p)
Asparagine	13.899	3.7969	0.00014601	3.8356
Vanillin	12.703	3.6671	0.000093316	4.03
Glutamine	7.9232	2.9861	0.0001551	3.8094
Glucoraphanin	6.7452	2.7539	0.0095792	2.0187
Glucoalyssin	6.3933	2.6766	0.0061787	2.2091
Gluconapoleiferin	6.0895	2.6063	0.0037529	2.4256
Glucoerucin	5.6652	2.5021	0.0020502	2.6882
Glucoberteroin	5.6449	2.497	0.0021316	2.6713
Quinic acid	5.4022	2.4335	0.000082618	4.0829
4-Hydroxyglucobrassicin	5.1831	2.3738	0.036719	1.4351
Kaempferol	5.0276	2.3299	0.000000063753	7.1955
beta-Alanine	3.0032	1.5865	0.000093316	4.03
Tryptophane	2.9651	1.5681	0.00085686	3.0671
Pyroglutamic acid	2.8035	1.4872	0.000094005	4.0268
Xylose	2.518	1.3323	0.00028982	3.5379
Glycine	2.5012	1.3226	0.000093316	4.03
Leucine	2.1608	1.1116	0.001145	2.9412
Serine	2.1444	1.1006	0.000084015	4.0756
Aspartic acid	2.0886	1.0625	0.0022432	2.6491
Glucoiberin	2.071	1.0504	0.021084	1.6761
Glutamic acid	2.0292	1.0209	0.0001551	3.8094
Gallic acid	0.31341	-1.6739	0.00018096	3.7424
Neoglucobrassicin	0.27359	-1.8699	0.0035157	2.454
Cyanidin 3-sinapoylsinapoyldiglucoside-5-glucoside	0.18844	-2.4078	0.000093316	4.03
Cyanidin 3-feruloylsinapoyldiglucoside-5-glucoside	0.1881	-2.4104	0.000094005	4.0268
Cyanidin 3-sinapoyldiglucoside-5-glucoside	0.18775	-2.4131	0.000093316	4.03
Cyanidin 3-caffeoylp-coumaroyldiglucoside-5-glucoside	0.18772	-2.4133	0.000093316	4.03
Cyanidin 3-feruloylsinapoyldiglucoside-5-glucoside.	0.18716	-2.4177	0.000093316	4.03
Cyanidin 3-glycopyranosyl-sinapoyldiglucoside-5-glucoside	0.18488	-2.4354	0.00013355	3.8743
Cyanidin 3-sinapoylglucoside-5-glucoside	0.18426	-2.4402	0.00017821	3.7491
Cyanidin 3-diglucoside-5-glucoside	0.1837	-2.4446	0.00017821	3.7491
Cyanidin 3-p-coumaroyldiglucoside-5-glucoside	0.18264	-2.4529	0.00019426	3.7116
Cyanidin 3-sinapoyldiglucoside-5-glucoside.	0.1825	-2.454	0.00018151	3.7411
Cyanidin 3-p-coumaroylsinapoyldiglucoside-5-glucoside	0.17709	-2.4974	0.001145	2.9412
Cyanidin 3-p-coumaroylsinapoyltriglucoside-5-glucoside	0.17329	-2.5288	0.0010831	2.9653
Glucobrassicanapin	0.16855	-2.5687	0.020637	1.6854
4-Aminobutyric acid	0.078828	-3.6651	0.000020389	4.6906
